# A pioneer of research on aureomycin synthesis and bacterial nitrogen fixation in China: Shen San-Chiun

**DOI:** 10.1093/procel/pwad001

**Published:** 2023-01-12

**Authors:** Huan Liu, Jianliang Huang, Wanying Gao, Hao Cheng

**Affiliations:** Department for the History of Science and Scientific Archaeology, University of Science and Technology of China, Hefei 230026, China; State Key Laboratory of Virology, Wuhan 430072, China; Department for the History of Science and Scientific Archaeology, University of Science and Technology of China, Hefei 230026, China; Department for the History of Science and Scientific Archaeology, University of Science and Technology of China, Hefei 230026, China; Institute of Microbiology, Chinese Academy of Sciences, Beijing 100101, China

Shen San-Chiun (1917–2021) (Fig. 1) is a well-known microbial biochemist and molecular geneticist in China. He was elected as a member (academician) of the Chinese Academy of Sciences in 1980. Since studying and returning to China, he has successively worked at Zhejiang University and Shanghai Institute of Microbiology, Chinese Academy of Sciences. Due to the changing needs during the development of the country, Shen San-Chiun resolutely changed his career, specializing in the research of aureomycin synthesis and bacterial nitrogen fixation. In the early 1950s, Shen San-Chiun proposed a method for the mass production of aureomycin, making China the fourth country capable of producing this important tetracycline-class antibiotic. In 1960, Shen San-Chiun’s team discovered that the substance in *Bacillus subtilis* that changes the genetics of penicillin is RNA (Shen San-Chiun et al., 1960). Since 1973, Shen San-Chiun turned to research on the structure and regulation mechanism of nitrogen-fixation genes, and in 1977, he published a paper denying the international authoritative view of “silent regions existing between genes” (Hsueh et al., 1977). It is commendable that Shen San-Chiun always adhered to his original intention, cared about the development of genetics in China, and cultivated many talents for the country. Ultimately, his work also led to the establishment of the State Key Laboratory of Plant Molecular Genetics in 1986, which made significant contributions to the development of molecular genetics in China (Xiong, 2014).

**Figure 1.**
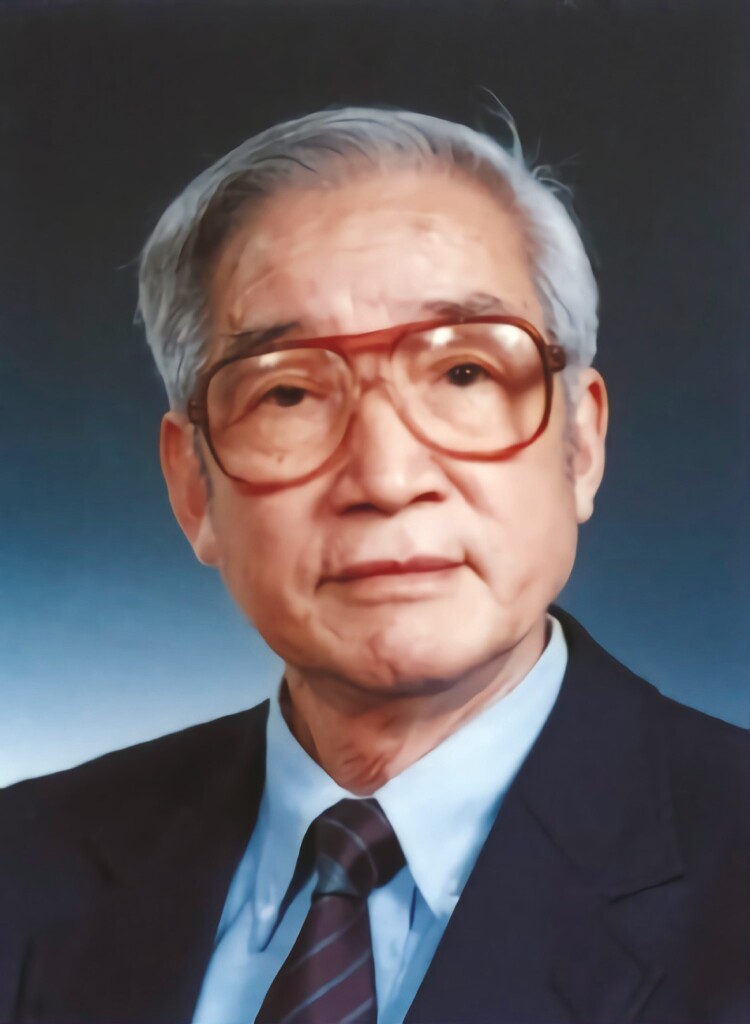
Shen San-Chiun.

## Study and work experience

Shen San-Chiun was born in Wujiang, Jiangsu, and his family had a long tradition of reading books. In 1931, Shen San-Chiun was admitted to Wujiang Middle School and later studied at Suzhou Agricultural School in Jiangsu Province. In 1937, he was admitted to Nanjing University. Unfortunately, at that time, East China was a war zone, and Shen San-Chiun had to move westward with the university, successively fleeing to Anhui, Hubei, and later Guangxi. During his time at the College of Agriculture, Guangxi University, under the guidance of Chang Chao-Chien and Yu Ching-Jang, Shen San-Chiun transferred to the National South-West Associated University in 1939, where he studied under Professor Chang Chin-Yuch, during which time he developed an interest in genetics. In 1947, Shen San-Chiun went to California Institute of Technology, the birthplace of “molecular genetics,” to study under the tutelage of Norman Horowitz, and received his Ph.D. in 1950 (Shen San-Chiun, 2009).

**Figure 2.**
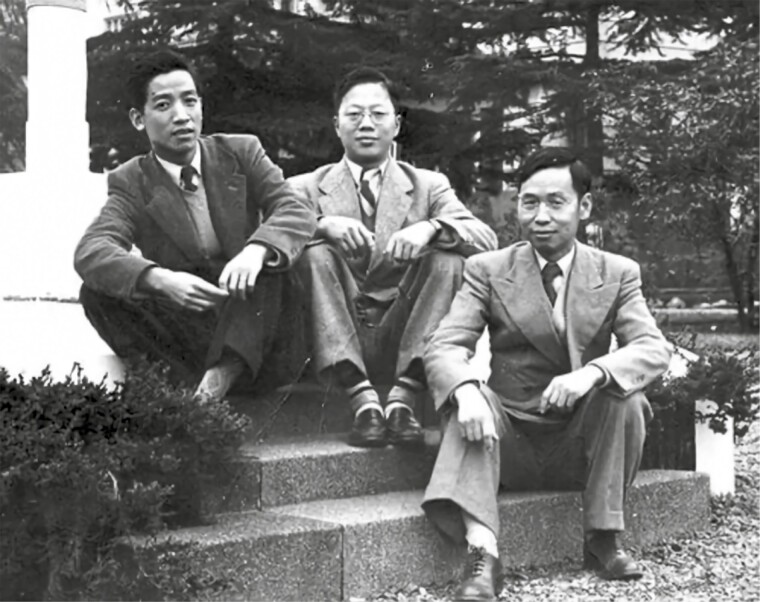
Shen San-Chiun (left), Lo Shih-Chun (middle), and Chao Chung-Yao (right) during detention in Japan.

On the way back from his studies, Shen San-Chiun was detained by the USA occupying forces in Tokyo, which greatly complicated his return to China (Fig. 2). Nevertheless, he remained true to what he was thought, kept in mind his responsibility to China, and finally overcame all difficulties and returned to the mainland. In the early days of returning to China, Shen San-Chiun taught at Zhejiang University and later transferred to the Plant Physiology Laboratory in Shanghai in 1952 (independently established as the Institute of Plant Physiology, Chinese Academy of Sciences in 1953), focusing on aureomycin (Shen San-Chiun, 2002). In 1978, Shen San-Chiun turned toward research on bacterial nitrogen fixation and opened a new field of bacterial nitrogen fixation in China. In 1981, Shen San-Chiun was formally elected as a member (academician) of the Department of Biology, Chinese Academy of Sciences.

## Achievements of aureomycin development in China

In the early 1950s, when foreign antibiotics were widely used and mass produced, domestic research on antibiotics in China was in its infancy. Since Duggar discovered aureomycin in 1945, the technology has been monopolized by the USA, the UK, and Italy, while the supply and demand of domestic antibiotics had become increasingly unbalanced. In 1952, the national authorities convened a symposium on antibiotic research and drew up a plan, which clarified the primary goal of mass production of penicillin, as well as the completion of the development and mass production of chloramphenicol, streptomycin, and aureomycin, while Shen San-Chiun was mainly responsible for research on the structure and synthesis mechanism of aureomycin (Niu, 2010).

Although Shen San-Chiun was not fully prepared for antibiotic research, he accepted Yin Hung-Chang’s invitation without hesitation and was assigned to the research on aureomycin (Shen San-Chiun, 2002). In 1953, Shen San-Chiun formally devoted himself to the key issue of strain breeding and aureomycin fermentation, with the aim to realize the mass production of aureomycin. Initially, the literature on the production technology of aureomycin was quite limited, so Shen San-Chiun led the team to the Shanghai No. 3 Pharmaceutical Factory, which produced penicillin, to gain experience with fermentation. In the same year, Shen San-Chiun found in an experiment that the initial growth and development environment of *Streptomyces aureofaciens*, including the nutrient composition and pH value of the initial medium, will significantly affect the production of aureomycin. The following year, the discovery was published in the *Journal of Experimental Biology*, which became a pioneering achievement of aureomycin research in China (Shen San-Chiun et al., 1954).

**Figure 3.**
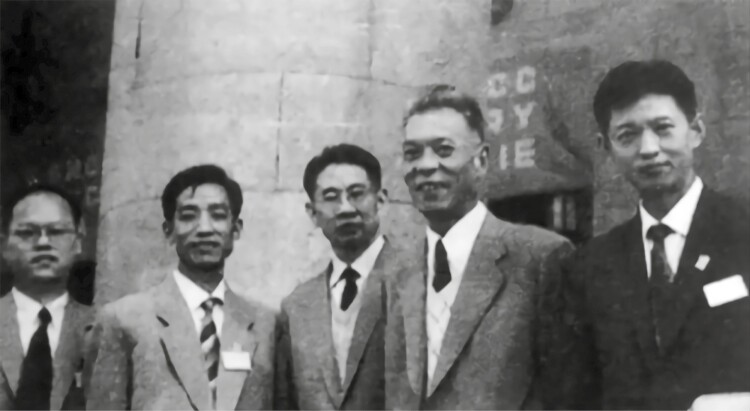
The Fifth International Biochemical Conference in 1961 (the second from the left is Shen San-Chiun).

In addition, in the research on the metabolism and synthesis of aureomycin, Shen San-Chiun’s team also took the lead in hypothesizing that the content of phosphate in the fermentation medium would directly affect the production of aureomycin. The results of aureomycin physiology research and mass production strategy were also published in the *Science Bulletin* in 1955 (Shen San-Chiun, 1955). In 1953, the research on aureomycin successfully passed the pilot production test of 53 gallons, and the effective antibacterial compound aureomycin hydrochloride in a 1-mg sample was as high as 1,600 units (1 μg is 1 unit), fully compliant with international standards. In 1954, the research results were translated to clinical trials, and the results showed that the pharmaceutical quality and performance of domestic aureomycin reached the international level (Fu et al., 1958) (Fig. 3).

## Genetics of bacterial nitrogen fixation

Answering the country’s needs, Shen San-Chiun opened a new research field in 1973—bacterial nitrogen fixation. Although Shen San-Chiun’s knowledge of biogenetics remained at the level it had been when he returned to China in the 1950s, he still rose to the challenge. To make up for the recent 20 years of international frontier research, he began to consult a large number of genetics literature. Finally, inspired by Valentine’s team at the University of California, Shen San-Chiun chose the genetic mechanism of bacterial nitrogen fixation as the starting point (Xiong, 2014).

**Figure 4.**
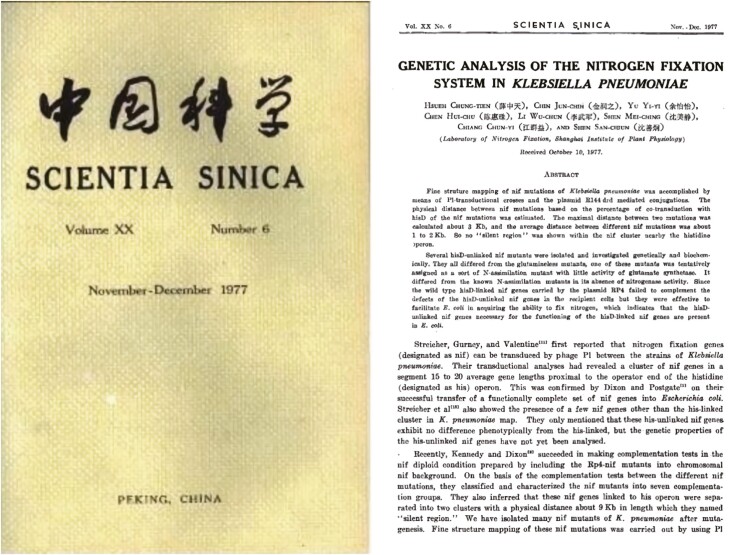
“Genetic Analysis of the Nitrogen Fixation System of Klebsiella pneumoniae” published in 1977.

**Figure 5.**
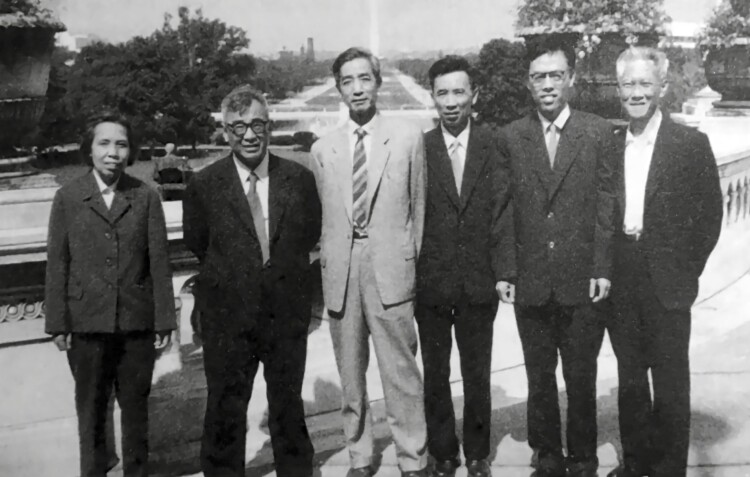
The international conference on bacterial nitrogen fixation in 1978 (the third from the left is Shen San-Chiun).

In 1977, Shen San-Chiun’s team not only discovered the nitrogen-fixation gene *nifC*, but also confirmed that it is arranged in a cluster on the chromosome of *Klebsiella pneumoniae* (Hsueh et al., 1977). This experimental result for the first time falsified the authoritative view that “silent regions exist between genes,” generally accepted internationally at that time, which not only promoted the research on the genomic structure of nitrogen-fixing genes, but also clarified important functional aspects of gene transcription. In the year of publication, this discovery attracted the attention of the international nitrogen-fixation research community, and gradually brought China’s nitrogen-fixation genetic research in line with international standards (Fig. 4). In 1978, Shen San-Chiun’s team put forward the hypothesis of two-level regulation of nitrogen-fixation genes, which postulated that the regulation of nitrogen-fixation genes by oxygen is based on the transcription level of the *nifA* gene and the activity level of NifA protein. In addition, Shen San-Chiun’s team also found that the dependence of the *nif* gene promoter on the NifA product is directly related to the activity of the conserved sequence of the promoter itself (Horowitz, 1979). This work immediately became an important milestone in the research on the structure and regulation of nitrogen-fixation genes. The team won the first prize in Natural Science of the Chinese Academy of Sciences in 1979, and the laboratory became one of the centers of international bacterial nitrogen-fixation research (Fig. 5).

## Pioneer of molecular genetics in China

After returning to China, Shen San-Chiun always put the country’s development needs above his personal pursuits and successively contributed to important issues such as the production of aureomycin and bacterial nitrogen fixation in the country. Although Shen San-Chiun failed to engage in his favorite molecular genetics research, it did not diminish his enthusiasm for genetics and his ambition to reinvigorate domestic molecular genetics.

**Figure 6.**
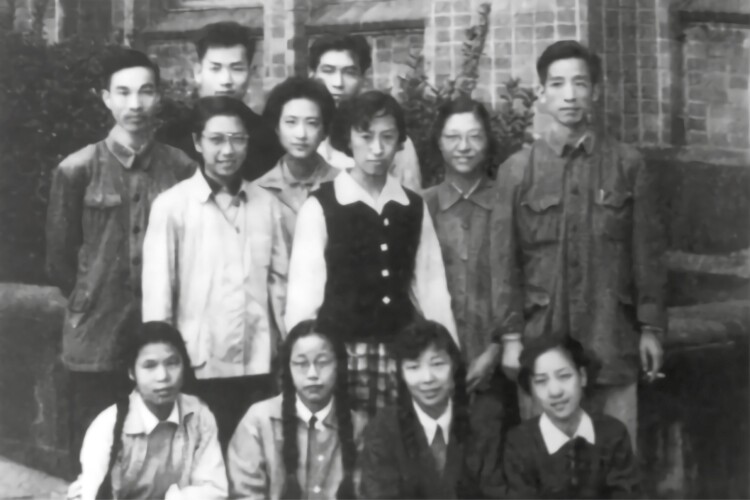
Shen San-Chiun with students and researchers at the Institute of Biotechnology (the first from the right in the middle row is Shen San-Chiun).

In 1960, Shen San-Chiun’s team discovered that there is a factor in *B. subtilis* that changes its genetic response to penicillin, and that it is not deoxyribonucleic acid, but ribonucleic acid (Shen San-Chiun et al., 1960). This research result proved that RNA can also carry genetic information, denying the international authoritative theory that DNA is the only basis of inheritance. This cutting-edge discovery brought China’s genetics research closer to the leading level of the world. In 1963, Shen San-Chiun’s team discovered a new metabolic pathway for hexose decomposition and speculated that the presence of NAD(P) nucleosidase would have a damaging effect on auxiliary enzymes (Wang et al., 1963). To varying degrees, this achievement had an important impact on the development of international genetics and also laid the groundwork for the revival of domestic molecular genetics in China. The conclusion of the genetic analysis of the nitrogen-fixation system published by Shen San-Chiun’s team in 1977 not only became one of the earliest publications of molecular genetics in China, but also had a significant impact on the international research field (Fig. 6).

In addition, Shen San-Chiun also attached great importance to the cultivation of talents. While employing accomplished researchers in the institutes, he also guided students from other universities to conduct research and recommended young talents to study abroad. In 1984, Shen San-Chiun proposed the establishment of the State Key Laboratory of Plant Molecular Genetics, which was realized 4 years later (Shen San-Chiun, 2009).

## One life for science and another for country

“In my eighties, I often thought that there were two purposes in my life, one for science and the other for our country,” Shen San-Chiun said in his later years. While devoting himself to science, Shen San-Chiun took the lead in using the principles of biological induction of aureomycin synthesis to cultivate strains with stronger vitality and higher antibiotic yield. In the study of bacterial nitrogen fixation, Shen San-Chiun’s team not only found that nitrogen-fixing genes are arranged in a cluster on the chromosome, but also found that RNA can change the genetic traits related to penicillin in *B. subtilis*. Despite the hard return journey, Shen San-Chiun retained a sincere patriotism and continued to break through the technical barriers toward the development and mass production of aureomycin to solve the urgent needs of the country. Shen San-Chiun was a pioneer of molecular genetics and bacterial nitrogen-fixation research in China, and has trained many professionals in his research field, exhibiting exemplary bravery while climbing the peaks of scientific discovery.
